# Laue-DIC: a new method for improved stress field measurements at the micrometer scale

**DOI:** 10.1107/S1600577515005780

**Published:** 2015-05-09

**Authors:** J. Petit, O. Castelnau, M. Bornert, F. G. Zhang, F. Hofmann, A. M. Korsunsky, D. Faurie, C. Le Bourlot, J. S. Micha, O. Robach, O. Ulrich

**Affiliations:** a LEME, Université Paris Ouest, 50 rue de Sèvres, F-92410 Ville d’Avray, France; b PIMM, CNRS, Arts and Métiers ParisTech, 151 Bd de l’Hopital, F-75013 Paris, France; c Laboratoire Navier, Université Paris-Est, École des Ponts ParisTech, F-77455 Marne-la-Vallée, France; d Department of Engineering Science, University of Oxford, Parks Road, Oxford OX1 3PJ, UK; e LSPM, CNRS, Université Paris 13, 93430 Villetaneuse, France; f INSA-Lyon, MATEIS CNRS UMR5510, F-69621 Villeurbanne, France; g Université Grenoble Alpes, INAC-SPrAM, F-38000 Grenoble, France; h CNRS, SPrAM, F-38000 Grenoble, France; i CRG-IF BM32 at ESRF, 71 Avenue des Martyrs, F-38000 Grenoble, France; j CEA, INAC-SP2M, F-38000 Grenoble, France

**Keywords:** X-ray diffraction, microbeam, stress field, elastic strain

## Abstract

A better understanding of the effective mechanical behavior of polycrystalline materials requires an accurate knowledge of the behavior at a scale smaller than the grain size. The X-ray Laue microdiffraction technique available at beamline BM32 at the European Synchrotron Radiation Facility is ideally suited for probing elastic strains (and associated stresses) in deformed polycrystalline materials with a spatial resolution smaller than a micrometer. However, the standard technique used to evaluate local stresses from the distortion of Laue patterns lacks accuracy for many micromechanical applications, mostly due to (i) the fitting of Laue spots by analytical functions, and (ii) the necessary comparison of the measured pattern with the theoretical one from an unstrained reference specimen. In the present paper, a new method for the analysis of Laue images is presented. A Digital Image Correlation (DIC) technique, which is essentially insensitive to the shape of Laue spots, is applied to measure the relative distortion of Laue patterns acquired at two different positions on the specimen. The new method is tested on an *in situ* deformed Si single-crystal, for which the prescribed stress distribution has been calculated by finite-element analysis. It is shown that the new Laue-DIC method allows determination of local stresses with a strain resolution of the order of 10^−5^.

## Abstract 2

The increment of elastic strain distribution, with a micrometer spatial resolution, is obtained by the correlation of successive Laue images. Application to a bent Si crystal allows evaluation of the accuracy of this new Laue-DIC method, which is about 10^−5^.

## 1. Introduction   

With the increasing need from industry to develop materials of high mechanical performance, a good understanding of the material properties at the microscale (0.1–10 µm) has become critical since many of these properties are responsible for the macroscopic (*i.e.* millimeter) mechanical behaviour. Many research efforts during the last decade have been focused on the characterization and understanding of the stress and *total* strain fields heterogeneities in deformed polycrystals at a fine scale. *Elastic* strain fields (and associated stress fields) with a submicrometer spatial resolution can be investigated, in principle, by the analysis of Kikuchi (Maurice *et al.*, 2011 ▸; Wilkinson *et al.*, 2006*a* ▸,*b* ▸) or Kossel (Morawiec *et al.*, 2008 ▸) diffraction patterns when acquired and analyzed with a sufficiently high resolution in a scanning electron microscope. Besides, third-generation synchrotron radiation facilities such as the ESRF in Grenoble (France) are able to produce very intense X-ray beams with submicrometer cross section. First attempts to use such a highly focused X-ray beam to investigate the stress field heterogeneity in deformed polycrystals, at an intragranular scale, used *monochromatic* beams; in that case, one needs to rotate the whole specimen about the grain to be measured, but, due to the sphere of confusion of goniometers, which is rarely better than 20 µm, a micrometer spatial resolution could not be achieved (Castelnau *et al.*, 2001 ▸; Ungár *et al.*, 2007*a* ▸). Nevertheless, one solution that has been proposed to account for the circle of confusion rotation problem using monochromatic beams employs high-resolution imaging of the diffracted beam (*e.g.* Hassani *et al.*, 2007 ▸).

Alternatively, this resolution issue can be solved by using a broadband *polychromatic* (or white) X-ray beam since, in that case, the specimen no longer needs to be rotated but just scanned (translated) in front of the beam. When the grain size is larger than the beam cross section and the X-ray penetration depth, a Laue pattern coming from a single (sub)grain can be acquired at each specimen position, making it possible to distinguish diffraction patterns related to different grains (or subgrains) with a typical probe volume of the order of few µm^3^. Consequently, heterogeneities of elastic strain (and associated stress) at the micrometer scale can be, in principle, characterized. Important applications using this technique to identify phase and strain with submicrometer spatial resolution can be found in the literatue (Chung & Ice, 1999 ▸; Barabash *et al.*, 2001 ▸; Tamura *et al.*, 2002*a* ▸; Mughrabi & Ungár, 2006 ▸; Levine *et al.*, 2006 ▸; Hofmann *et al.*, 2013 ▸).

Laue diffraction patterns are typically recorded on a two-dimensional detector; local lattice orientation and local elastic lattice strain can then be deduced from the position of at least four Laue spots on the detector. Specific software such as *XMAS* (XMAS, 2003 ▸; Tamura *et al.*, 2002*b* ▸), *LaueGo* (LaueGo, 2010 ▸) and *LaueTools* (Lauetools, 2010 ▸), mostly based on the calculations presented by Chung & Ice (1999 ▸), have been developed for that purpose, and have been made available to the community. They allow a rapid indexing of Laue spots and the calculation of the orientation and deviatoric strain tensors. In spite of the quality of these analysis routines, some uncertainties may be introduced in the estimation of local strain, since (i) the determination of the Laue spots position relies on their fit by Gaussian- or Pearson-type functions, that are sometimes not appropriate, and (ii) the evaluation of the absolute spot position strongly depends on geometrical features of the experimental setup which must be known to a high accuracy. Consequently, uncertainties on the orientation-strain matrix are often too large to allow their use for micromechanical studies (Hofmann *et al.*, 2011 ▸). For example, reaching a 10 MPa uncertainty on stress measurement for steel (equivalent to a 5 × 10^−5^ uncertainty on strain) typically requires determining the diffracted beams’ direction with an angular accuracy of 0.1 mrad which corresponds to an accuracy of ∼0.1 pixel on spot position with the setup configuration routinely used at beamline BM32. Such a resolution cannot be reached if the spot shape deviates from Gaussian- or Pearson-type.

The aim of this paper is to present a new method (called Laue-DIC) in which Laue spots do not need to be fitted with an analytical function. Uncertainties due to geometry errors are minimized, and it becomes possible to determine strains with much improved accuracy. The displacement of each Laue spot is investigated without the requirement to determine its position with high accuracy; this is realised by applying the Digital Image Correlation (DIC) technique (Sutton *et al.*, 2009 ▸; Bornert *et al.*, 2012 ▸) to Laue patterns recorded at different positions of the probe volume. Thanks to the high accuracy of DIC that can be of the order of a few hundredths of a pixel (Bornert *et al.*, 2009 ▸), we show that local strain can be measured with a resolution as good as 10^−5^.

The paper is structured as follows. We provide a short overview of the microdiffraction beamline BM32 at the ESRF in §2. The problem formulation is presented in §3. In §4 we provide an estimate of the accuracy of DIC applied to Laue images. Finally, in §5, to illustrate the potentiality of the method, we consider the case of a Si single-crystal deformed under four-point bending. Elastic strain profiles across the specimen are obtained at a given loading step by performing a line-scan across the sample surface with the white micro-beam, with micrometer spatial resolution, recording a Laue pattern at each beam position. The deviatoric stress tensor is calculated using the anisotropic elastic constants, and experimental results are compared with finite-element (FE) calculations of the deformed crystal. Results are presented in terms of stress, firstly to compare the resolution obtained by Laue-DIC with the applied stress and material properties, like yield stress; and, secondly, the stress analysis allows the surface free stress condition and the bending moment value to be verified.

## 2. Microdiffraction setup at BM32   

The usual way of performing X-ray diffraction on single crystals is to set the photon energy (inversely proportional to the wavelength λ) and map the Bragg reflection peaks by rotating the sample while detecting the diffracted X-rays with a detector. The Bragg law,
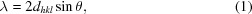
with θ the scattering angle, allows an estimation of the mean lattice spacing 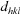 of the diffracting planes with Miller indices 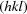. This technique becomes inappropriate for very small beam and high spatial resolution as fine as a micrometer, since current high-quality diffractometers exhibit a sphere of confusion (*i.e.* the distance between all needed rotation axes) of a few tens of micrometers at best, and thus any sample rotation would move the point of interest in the sample out of the microbeam (Castelnau *et al.*, 2001 ▸; Ungár *et al.*, 2007*b* ▸).

With the microdiffraction setup available at beamline BM32 at the ESRF, the sample does not need to be rotated thanks to the use of a white X-ray beam, and thus the spatial resolution is only limited by the beam size and the penetration depth. A detailed description of the experimental setup can be found by Ulrich *et al.* (2011 ▸). Briefly, the white beam generated by the bending magnet, with a relatively flat spectrum ranging between 5 and 22 keV, is focused down to a submicrometric cross section, around 1 µm × 1 µm for the experiment presented hereafter, by a pair of Kirkpatrick–Baez mirrors. The beam position being constant and very stable, diffraction Laue patterns are obtained by simple translation motions of the sample in front of the beam. For each sample position, the diffracted X-rays are recorded on a two-dimensional detector. Here, we are considering experiments performed with a MAR165 CCD detector; it is made up of a scintillator linked to a CCD sensor by a single fiber-optic taper and the demagnification ratio is 2.7:1. The CCD is a 4096 × 4096 pixels binned 2 × 2 sensor with a pixel size of 80.6 µm and a saturation level of 360000 electrons for 12 keV photons. Typical exposure time was 0.5 s and images are digitized with a 16-bit A/D converter with a readout time of about 5 s.

**Figure 1:**
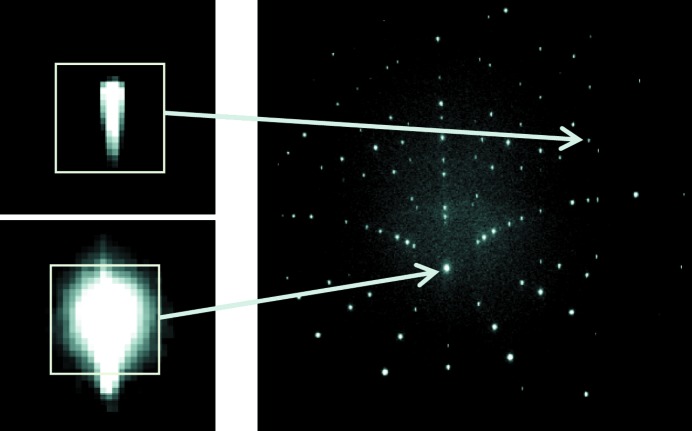
Typical Laue pattern obtained on a Si single-crystal. The light gray squares represent the zone of interest (ZOI) around Laue spots (zoom) used for DIC.

A typical Laue image obtained from a Si single-crystal is shown in Fig. 1 ▸. It consists of over 100 Laue spots with an elongated shape due to the penetration of X-rays into the thick Si crystal. We observe that the higher the spot intensity, the more closely the top of the spot approaches a Gaussian shape, but none of them can be really fitted by a Gaussian with high accuracy. The rest of the image is formed by background noise, mostly due to diffuse scattering and fluorescence. The background is very slightly domed from the center of the image to the detector periphery.

## 3. Problem formulation   

In this section we describe how the elastic strain, and its accuracy, can be obtained from Laue images. We recall that in white-beam Laue microdiffraction, only angles between diffraction vectors are measured and not the lattice spacings, 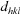, because of the impossible acccess to the hydrostatic part of the deformation tensor by this technique. Nevertheless, lattice orientation, angles between the lattice vectors and length ratio between these vectors can be determined.

Let us consider two configurations (or deformation/orientation states) for the specimen, a reference configuration and a deformed configuration. These two configurations can, for example, correspond to two Laue measurements at the same spatial position on the specimen but for two different loading states, when the specimen is deformed *in situ*. Hereafter, the two configurations will correspond to two different positions of the X-ray beam on the deformed specimen (single-crystal), at a given loading state; since the specimen is deformed heterogeneously (by bending, see §5), the two positions correspond indeed to two different elastic strains.

We consider matrices whose columns are the components of the three lattice vectors 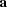, 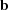, 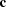 of the crystal, expressed in an orthonormal reference frame. We denote in the following 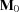 the matrix corresponding to the reference configuration, and 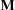 that for the deformed configuration. The mechanical transformation gradient 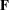, between the reference and deformed configurations, relates matrices 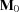 and 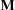 in the following way,
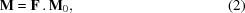
where the dot ‘.’ expresses the scalar product, *i.e.* the above equation reads 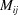 = 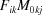 in which summation over the repeated index *k* is implicit (Einstein convention), or, equivalently, 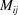 = 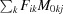. In the general case, 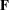 has nine independent components, but, since lattice dilation can only be measured using a monochromatic beam (see, for example, Robach *et al.*, 2013 ▸), only eight components of 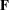 can be evaluated with white-beam Laue microdiffraction; the trace of 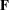 remains undetermined and only the deviatoric strain tensor can be obtained. Within the general finite transformation framework, 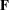 can be uniquely decomposed into the product of an (orthogonal) rotation tensor, 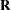, and a (symmetrical right Cauchy–Green) strain tensor, 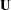, from which the Green–Lagrange strain tensor, 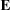, can be extracted,
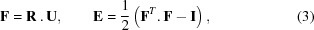
with 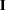 the (second-order) identity tensor and 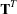 the transpose of 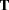.

We also define a geometrical function *f* that relates the position 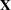 of a given Laue spot on the detector (*i.e.* the spot coordinates in a two-dimensional reference frame attached to the detector screen) to the Miller indices 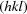 of the corresponding diffracting plane. Denoting 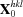 the Laue spot coordinates for the reference configuration and 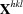 those for the deformed configuration, we have
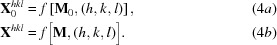
Here, function *f* accounts for the complete geometrical arrangement of the setup (sample-to-detector distance, detector orientation, *etc*.). Setup parameters are defined in Appendix *A* [App appa] and the expression for function *f* is detailed in Appendix *B* [App appb].

### 3.1. Standard procedure for Laue microdiffraction   

The standard procedure classically used for the estimation of elastic strain from Laue patterns (as in *XMAS* and *LaueTools* software) runs as follows. First, all geometrical parameters (detector position and beam orientation) entering in function *f* are evaluated with a well known and strain-free specimen, such as a Ge single-crystal. Next, the Laue pattern of the specimen of interest is measured using the same geometrical setup. Positions 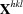 of Laue spots on the two-dimensional detector are estimated with a fitting of the measured spots by standard analytical functions such as Gaussian- or Pearson-type. Knowing the Miller indices for all available spots, the set of available relations, equation (4*b*) (one per spot), is then inverted to find 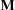. Finally, the transformation gradient 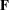 is evaluated with relation (2) in which the undeformed lattice parameters entering in 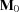 are usually taken from the literature. Four main sources of uncertainties thus arise:

(i) The function *f* is obtained from the reference Ge crystal by minimizing an error function (in a least-square minimization sense) associated with the distance between the measured positions **X**_Ge_(*hkl*) of Laue spots on the detector with the 206 computed ones *f*[**M**_Ge_, (*h*, *k*, *l*)], for all (*hkl*) reflections. Some inaccuracies arise here since the measured positions are sensitive to distortions of the detector grid. Furthermore, spot positions are obtained by a Gaussian- or Pearson-type fitting. Typically, theoretical Ge spot positions match on average the measured positions with an accuracy of about two tenths of a pixel.

(ii) Since the penetration depth of the measured sample is generally different from that of the Ge calibration crystal, the mean scattering volume lies at a different position along the beam direction, compared with Ge. In particular for Si, overall attenuation (for all photon energies in the range 5–22 keV) is lower than for Ge. Thus, the calibration of the geometry determined from Ge will never be quite right, unless the investigated sample is also Ge. The uncertainty due to this effect is amplified when the detector is close to the sample. We also note that each spot has its own probing depth. To cancel out this effect and obtain better accuracy on differential strain inside a two-dimensional map, experimental geometry is sometimes calibrated using a Laue pattern from the real sample (*e.g.* at the center of the map).

(iii) Positions **X** ^*hkl*^ for the specimen of interest are determined by fitting the Laue spots with Gaussian- or Pearson-type functions. As illustrated in Fig. 1 ▸, such functions are not appropriate in many cases for reaching the required subpixel resolution.

(iv) 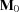 and 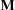 are determined independently. Strain-free lattice parameters entering in 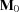 are usually taken from textbooks and may thus deviate from those of the actual specimen. Matrices 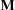 and 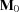 fully integrate the errors defined above on *f* and on Laue spot positions 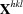 and 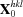, respectively. So, when multiplying the deformed state 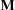 by the reference one 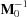 to find 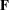 according to (2), errors on the geometrical calibration of the setup (that are included in the definition of *f*) and on spot positions are fully passed to the uncertainties on the tranformation gradient 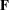.

For example, Magid *et al.* (2009 ▸) found stress fluctuations of the order of a GPa in a single-crystal of pure Cu, a result which might not be physically relevant. Error sources described above can be evaluated quantitatively as follows. Inverting equation (2),
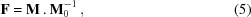
the uncertainty on 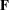, denoted 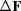, reads
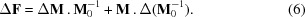
Expressing 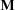 and 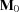 in nanometers so that their components are of the order of 1, 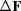 is of the same order of magnitude as 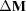 and 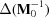. The uncertainty 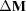 on 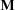 can hardly be better than a few 10^−4^ times the lattice spacing for the reasons explained above. Uncertainties on 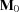 are generally of the same order for standard alloys as stress-free lattice parameters are difficult to be defined precisely. Hence, important uncertainties can arise in the determination of the deviatoric strain.

### 3.2. New Laue-DIC method for strain increments   

With the new approach proposed in this paper, we are characterizing the spot *displacement* 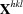 − 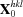 instead of the absolute positions 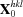 and 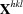 of the Laue spots. An accurate determination of 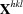 − 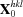 can be obtained by using the DIC technique between selected areas of two Laue patterns corresponding to each configuration; hence the Laue-DIC method.

Denoting 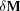 = 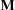 − 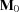, and restricting this error analysis (for sake of simplicity, in this specific section) to cases in which the two configurations are distinct by only small (elastic) strains and small lattice rotations (*i.e.* small displacements of Laue spots on the detector), 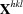 − 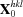 can be expressed with good accuracy by the first-order expansion of function *f*,
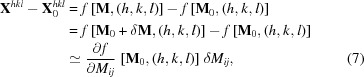
with implicit summation over indices *i* and *j*. To determine the eight independent components of 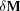, at least four independent couples 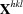 − 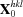 are needed. The inversion of equation (7) is possible since the inverse of matrix 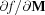 can be computed analytically. In this study, 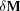 is then obtained by least-square minimization from the displacement of about 50 spots. The transformation gradient between the reference and deformed configurations is given by
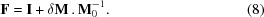
In doing so, uncertainties can be significantly reduced compared with the standard procedure, since one does not need to know very precisely the absolute spot positions 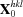, subjected, for example, to errors due to grid distortion of the detector, but only the relative motion of spots. More precisely, the uncertainty on 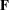 now reads
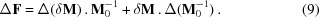
Compared with equation (6), the above estimation of 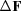 is several orders of magnitude smaller. As will be shown below, DIC allows estimating 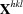 − 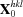 with an accuracy of a few hundredths of a pixel, and independently of the shape of Laue spots, leading to uncertainties on 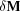 of the order of 10^−5^ times the lattice spacing. This feature originates from the fact that the gradient of *f*, 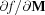, is much less sensitive to the precise value of 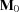 than the function *f* itself in equation (4). The last term on the right-hand side in equation (9) multiplies the increment of lattice parameter (generally ∼10^−4^) with the uncertainties on 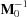 (say 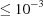). Consequently, highly accurate evaluations of local strain can be expected with the new Laue-DIC method.

## 4. Applying DIC to Laue patterns: procedure and performances   

### 4.1. DIC procedure   

DIC is a full-field measurement technique developed at the beginning of the 1980s (Sutton *et al.*, 1983 ▸, 1986 ▸; Bornert *et al.*, 2012 ▸). The method consists of matching a speckled pattern in similar images taken in the initial and deformed configurations, and provides a measurement of the displacement field of the pattern in the camera reference frame. In the experimental mechanics community, DIC is often used to measure the physical displacement field at the surface of the specimen itself. In that case, an artificial texture with a random pattern is deposited on the specimen surface (such as paint droplets) which is directly imaged with the camera sensor; the pattern generally has to be adapted to the investigated material and required spatial resolution. In our case, DIC allows measuring the displacement of the Laue spots on the detector screen. The speckled pattern is then directly provided by Laue spots, *i.e.* the image quality is essentially fixed.

The CCD camera pixel number, the dynamic range of the sensor and the signal-to-noise ratio influence the amount and the quality of information. Displacement resolution is often improved when there is a high dynamic range in the picture. Various error regimes have been identified (Doumalin, 2000 ▸; Bornert *et al.*, 2009 ▸), for which the dependence of the DIC accuracy and uncertainties on the speckle pattern and the parameters of the algorithms, such as sub-image size, gray-level interpolation method or shape functions (*e.g.* simple translation, translation + rotation with or without deformation), are discussed. For the present application, an obvious advantage of DIC is that it does not require any fitting of Laue spots by an analytical function; basically, DIC works for any spot shape, as long as there is still some similarity between spots before and after loading. Generally in polycrystals, this is true in elasticity, but Laue spots features can change a lot when plastic deformation appears (Castelnau *et al.*, 2001 ▸; Barabash *et al.*, 2002 ▸). In this study, we used the DIC software *CorrelManuV* developed at laboratories LMS-X (Palaiseau, France) and Navier (Marne-la-Vallée, France) (Bornert *et al.*, 2010 ▸).

To determine a displacement field 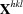 − 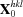 in an image of the deformed configuration with respect to a reference image, one considers a set of sub-images that will be referred to as the ‘zone of interest’ (ZOI). Each ZOI has a rectangular shape and is centered on one Laue spot. The ZOI size has been adapted to the spot dimensions: the ZOI was taken sufficiently large to encompass a whole Laue spot, but not too large to not encompass too much of the background signal. Here, we defined it as the smallest rectangle containing all pixels around the Laue peak having an intensity larger than a given threshold (see Fig. 1 ▸) fixed for all spots. The threshold is set only slightly larger than background noise, leading to an average signal to background-noise ratio per ZOI larger than 50.

The aim of DIC is to locate the same ZOI in two images captured at different positions **x** on the specimen, corresponding to two different local stress levels/orientations. The displacement of the center of a given ZOI between the two images is the displacement 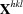 − 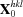. A correlation coefficient, compatible with a possible variation of brightness and contrast of gray level between both images, is used to measure the similarity between the sub-images. It is defined as
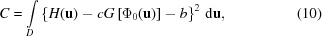
where *b* can be adjusted for compensating a possible offset of the brightness, and *c* for canceling effects due to the scale variations of intensity (*i.e.* exposure time) between both images. The functions 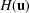 and 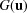 provide the intensity at an image point with coordinates 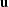 for the initial and deformed configurations, respectively. Finally, 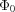 is the so-called ‘shape function’; it describes the distortion of the ZOI in the deformed configuration compared with the reference image. The shape function can include complex and inhomogeneous image distortions. For this very first application of Laue-DIC, we are dealing with data for which Laue spots are moving on the detector area, due to the heterogeneity of elastic strain, but their shape can be considered constant (see below). Therefore, we consider the most simple shape function, *i.e.* simple displacements of spots with no rotation nor shape change (*i.e.* a rigid-body translation), leaving only two degrees of freedom (displacement along *x* and *y* on the detector) for 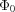. The optimization (or minimization) of the correlation coefficient *C* with respect to *b*, *c* and the parameters of 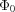 provides the best fit between both ZOIs, and the desired displacement is obtained. The interpolation of gray levels in the reference sub-image enables a subpixel accuracy for the displacement to be reached. In the following, bilinear, bicubic and biquintic interpolations have been tested.

### 4.2. Estimation of image noise   

**Figure 2:**
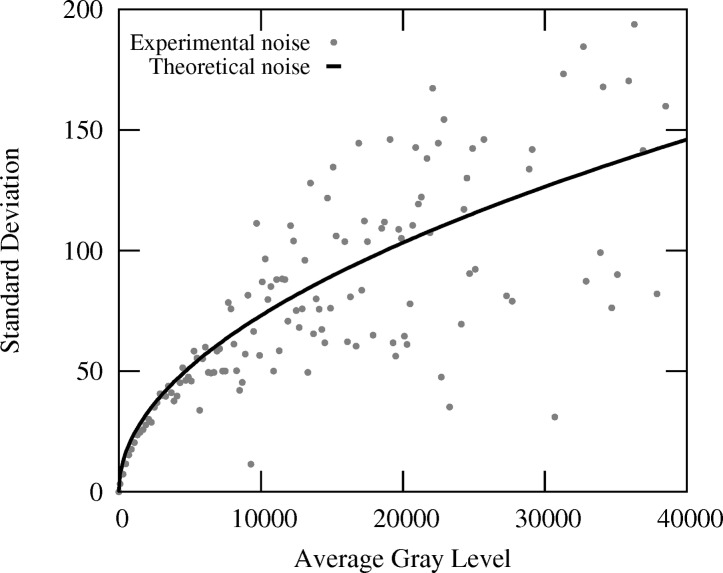
Correlation between the standard deviation of gray level (or intensity) and the average gray level, evaluated for a set of Si Laue patterns acquired under exactly the same conditions. Each point corresponds to a different pixel of the image. Theoretical noise = 0.73 × (average gray level)^1/2^.

The accuracy of elastic strain measurements can be affected by many features, as introduced above. In addition to uncertainties associated with possible errors and fluctuations of the geometrical calibration of the experimental setup (Hofmann *et al.*, 2011 ▸; Poshadel *et al.*, 2012 ▸), the image noise has to be considered. Here, the signal-to-noise ratio of typical Laue patterns for a Si single-crystal has been analyzed. We have considered a large number of Laue patterns measured in a row under exactly the same conditions, *i.e.* without changing the beam, specimen or detector position. For each pixel of the set of images, the average intensity and the standard deviation of the gray level have been extracted. Results are plotted in Fig. 2 ▸. The standard deviation of intensity, which is representative for the image noise, is found to be proportional to the square-root of the average gray level. The proportionality coefficient can be predicted as follows, assuming that photon noise is the sole contributor. The gray level *I* measured on an image pixel is proportional to the number *N* of photons received by the 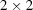 binned pixels (configuration used in this experiment), *I* = 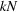. The coefficient *k* is the product of three terms,
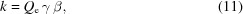
the quantum efficiency 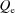, the electron/absorbed photon conversion rate γ (gain), and the gray-level/electron conversion rate β. The standard deviation of the intensity thus reads 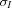 = 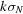 with 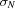 the standard deviation of the photons number, which is 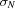 = 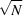 due to the Poisson distribution of *N*. Since 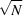 = 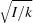, one thus obtains
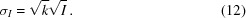
For the used MarCCD detector, according to the manufacturer, 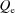 = 0.8, 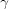 = 6 electrons per X-ray photon and 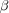 = 0.11 gray level per electron, for a photon energy of 12 keV. Consequently, one obtains a proportionality coefficient 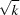 ≃ 0.73 which well matches the data of Fig. 2 ▸. From this analysis, it can also be concluded that photon noise largely dominates over other noise sources (dark noise, readout noise, *etc*).

### 4.3. Accuracy for subpixel displacements of Laue spots   

**Figure 3:**
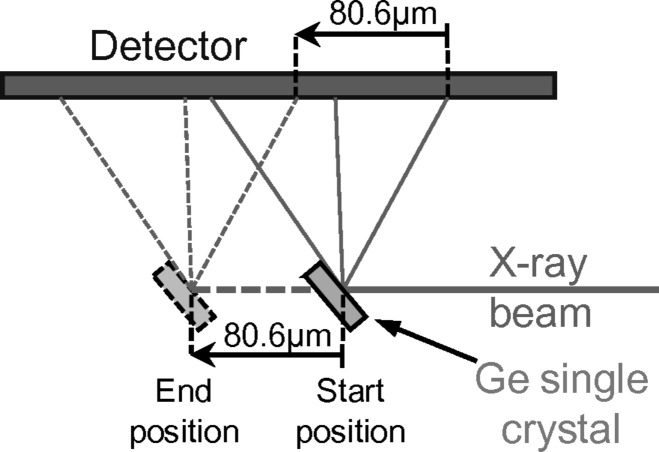
A Ge single-crystal is displaced along the incident X-ray beam for evaluating DIC accuracy for subpixel Laue spots displacements.

Before going to the application, it is important to estimate the accuracy and the resolution that DIC can achieve when the speckle pattern is the intensity distribution of a Laue spot. For this, successive Laue patterns were acquired on a Ge single-crystal that was translated in a direction (almost) parallel to the X-ray incident beam, *i.e.* (almost) parallel to the detector surface, as illustrated in Fig. 3 ▸. The distance covered by the Ge crystal (80.6 µm) matches the size of one pixel of the detector screen, and 100 Laue patterns were recorded at regular intervals during the Ge displacement. One might thus expect that these patterns are solely shifted from each other by an amount equal to the specimen translation. Looking at the difference between the spot displacements measured by DIC and that prescribed to the specimen provides an estimation of the DIC accuracy.

**Figure 4:**
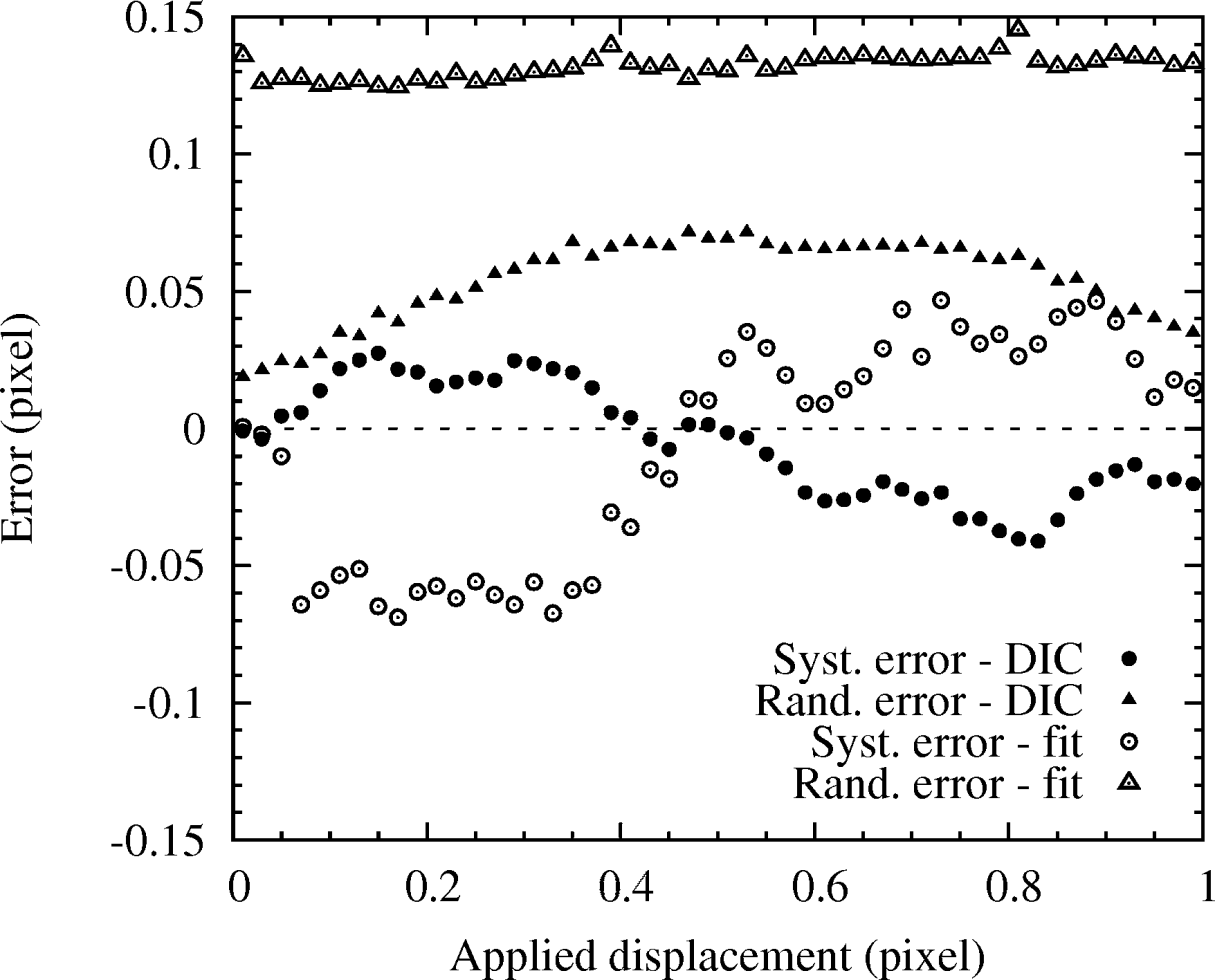
Systematic and random errors resulting from the DIC over 85 spots, obtained by subpixel translation of the specimen in a direction parallel to the detector surface. Both specimen positions and errors are expressed in pixel units.

Two types of error can be derived from this analysis (Fig. 4 ▸). The so-called ‘*systematic* error’ is the difference, expressed hereafter in pixel units, between the average displacement of all Laue spots of a given image and the prescribed sample displacement. It provides a measure of the overall displacement error resulting from the DIC technique. The so-called ‘*random* error’ is the standard deviation of the displacements measured for all Laue spots of a given image. Indeed, DIC does not guarantee that all spots are translated by the same amount; the *random* error provides an estimation of the displacement fluctuation. Results shown in Fig. 4 ▸ have been obtained using a biquintic gray-level interpolation on 85 spots and an intensity threshold of 75 to define the ZOI (for comparison, the maximum intensity of Laue spot is generally larger than 2000). Bilinear and bicubic interpolations provide very similar results. The *systematic* error often follows an S-shape curve (Bornert *et al.*, 2009 ▸), with here maximum values of ∼0.03 pixel and an average (of the absolute value) of 0.018 pixel. Best accuracy is obtained for image shifts of 0, 0.5 and 1 pixel. The *random* error is slightly larger; the maximum error is 0.07 pixel and its average is 0.054 pixel. Consequently, DIC allows the shift of *individual* Laue peaks to be estimated with an accuracy better than 0.1 pixel. This is enough to reach a stress resolution of the order of 1 MPa for a deformed silicon specimen, as illustrated below.

For comparison, the same data were processed with the standard method in which each peak was fitted by a Gaussian 2D function (Fig. 4 ▸). Spot displacement was then evaluated by comparison with the spot position obtained by Gaussian fitting of the initial image. Although the fitting was very good for this undeformed Ge single-crystal, both *systematic* and *random* errors are about twice as large as for Laue-DIC.

## 5. Application: strain and stress distribution in a bent Si crystal   

### 5.1. *In situ* mechanical test: setup   

**Figure 5:**
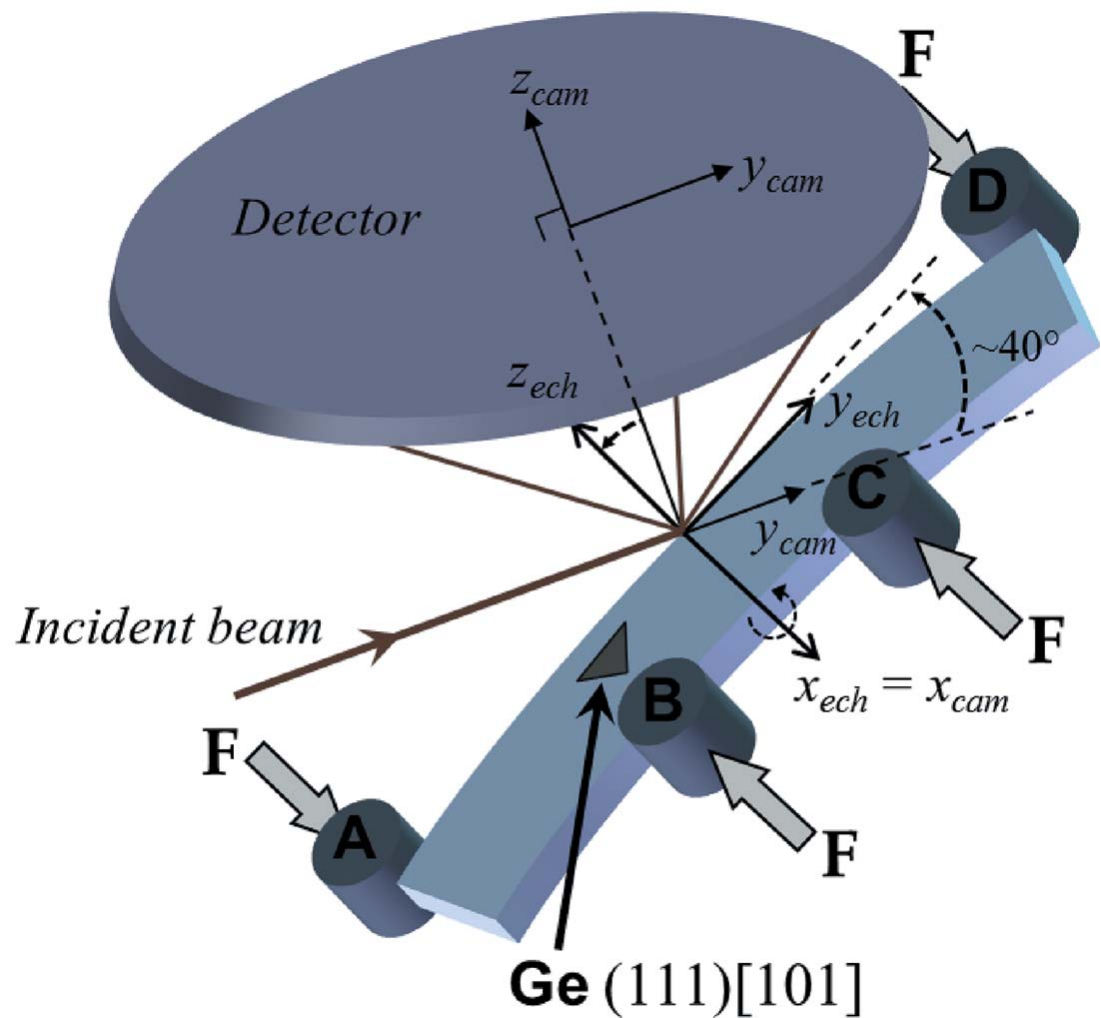
Schematic sample arrangement for *in situ* four-point bending measurements. The Si sample is scanned along direction 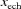, and at each position a Laue pattern is recorded on the CCD detector. A Ge single-crystal is used to calibrate the experimental geometry. The index ‘cam’ stands for the detector frame and the index ‘ech’ for the sample frame. Axis 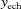 lies along the specimen length (longitudinal direction), 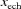 along the specimen width (transverse direction), which is also the loading direction, and 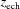 along the bending axis (normal direction).

Four-point bending tests were carried out on a Si single-crystal bar of length 10 mm (Fig. 5 ▸). The width of the bar (along direction 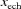) was 1.820 mm with a greatest deviation of ±1 µm over the 10 mm length. The thickness of the bar (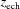 direction) varied linearly from 0.671 mm at one end to 0.683 mm at the other (Hofmann *et al.*, 2011 ▸). The crystal was oriented so that direction 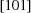 was approximately aligned with the sample 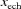 axis, 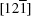 with 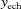 and 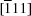 with 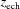. Flatness of all faces was better than 1 µm and the surfaces were polished to a mirror finish with negligible roughness. Loading was applied according to the schematic diagram in Fig. 5 ▸. The distance between loading pins A and D was 8 mm, and the distance between B and C was 3 mm. The sample was approximately tilted by 40° with respect to the incident X-ray beam. Laue patterns were recorded along a line parallel to the 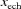 direction and centered between pins B and C (corresponding to 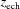 = 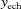 = 0 mm).

At the beginning and at the end of each loading step and Laue measurements, calibration patterns were collected on a Ge single-crystal positioned next to the scanned line. Ge Laue spots are very small and sharp, and therefore these patterns allow the accurate determination of all geometrical parameters of the experimental setup such as the detector-to-sample distance, the detector orientation, *etc.* (Appendix *A* [App appa]).

The four-point bending configuration is suitable for the study of the tensile and compressive material response. In the central area of the sample (between pins B and C), pure bending is expected. Kinematics and elasticity theory tell us that strain varies linearly along the transverse 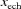 direction if the aspect ratio of the specimen is large enough (beam theory). As shown below, slight deviations from linearity will be observed in the present case. The sample was loaded incrementally; here, we report results obtained for three load levels, 0 N, 25 N and 50N.

### 5.2. Displacement of Laue spots from DIC   

**Figure 6:**
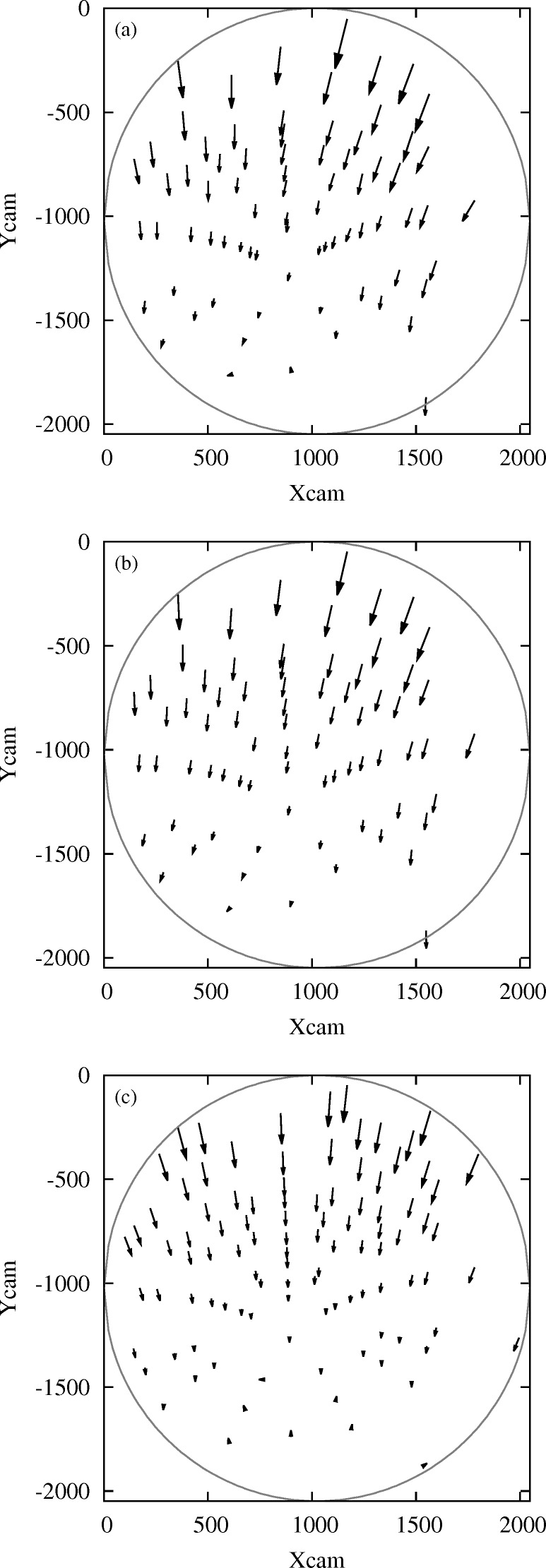
Laue spots displacement field between an end fiber at 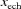 = 1.82 mm and the neutral fiber 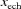 = 0.91 mm of the four-point bent Si crystal, at a loading of 50 N. (*a*) Experimental data analyzed by digital image correlation. (*b*) Theoretical field calculated after having estimated the corresponding transformation gradient 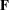. (*c*) Calculated field assuming uniaxial tensile stress. The scale of arrows has been enlarged by a factor of 75. The circle represents the active detector edge. Here, the detector axis 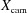 lies (approximately) parallel to the specimen loading direction, and 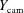 is (approximately) parallel to the incident X-ray beam. Both 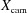 and 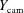 are given in pixel units. Low-intensity spots have been filtered out. Note that more spots appear in (*c*) than in (*a*) and (*b*) since theoretical patterns include all spots in the energy range 5–22 keV whereas low-intensity high-index spots have been filtered out from experimental data.

The deviatoric elastic strain tensor was evaluated from all the indexed Laue spots with maximum pixel intensity larger than 100 (gray level). Indexing was performed using the *LaueTools* software (Lauetools, 2010 ▸). DIC was performed between the Laue pattern measured at a given position 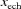 and the reference pattern measured at the position 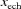 = 0.91 mm corresponding to the neutral axis of the Si beam. The motion of Laue spots between these two images (or configurations) provides the elastic strain and orientation distribution along the specimen width. As an illustration, Fig. 6(*a*) ▸ shows the spot displacement field on the detector area obtained by DIC for the 50 N load level. A collaborative movement of Laue spots towards negative 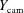 is observed.

Displacement 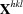 − 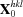 of Laue spots can also be calculated for any transformation gradient using equations (2) and (21). The effect of each individual component of 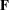 on the distortion of the Laue pattern has been investigated in detail by Petit *et al.* (2012 ▸). For the considered bending experiment, as deviatoric strains and rotations are both of the order of 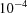 (see hereafter), the transformation gradient 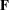 can be very well approximated within the simpler framework of small strain and small rotation, *i.e.*

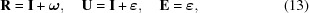
with 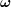 and 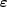 the infinitesimal rotation and strain tensors, respectively. Consequently, at first order,
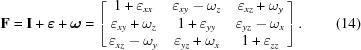
With this approximation, 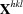 − 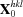 can be approximated as in equation (7). Fig. 6(*c*) ▸ shows the calculated displacement of spots assuming that the investigated volume element has been subjected to an uniaxial tension of 167 MPa along direction 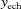 (corresponding to the 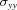 stress on an external fiber in our Si specimen at 50 N), as expected for pure bending with an undeformed neutral axis. It can be remarked that the shape of the experimental displacement field is very close to the theoretical one. However, some slight differences can be noticed. The origin of the remaining differences is not fully elucidated:

(i) Small shear stresses could arise because the bending direction is not aligned with the crystal symmetry axes, and the crystal is elastically anisotropic.

(ii) Slight imperfections of the bending test are also possible, *e.g.* small torsional loading and/or bending in a second direction could superimpose to the main loading direction, due to slight imperfection of the bending device and/or the sample mounting.

Small contributions of other strain components may therefore come into play in the experimental pattern.

### 5.3. Strain and stress profiles along the specimen width: main components   

As illustrated above, the actual transformation gradient 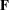 was adjusted for a closer match to experimental observations. The identification procedure used here consists of finding the eight independent components of the deviatoric part of 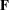 that best transforms 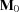 into 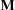 = 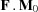 according to (8). The cost function is directly related to the spot displacements. The general expression to minimize reads
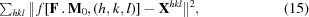
with 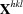 = 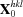 + 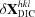 and 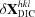 the displacement obtained by DIC. This minimization is performed using the Levenberg–Marquardt algorithm, considering all indexed spots with sufficient intensity. An excellent agreement is now obtained between the theoretical displacement field (Fig. 6*b* ▸) and the one measured by DIC (Fig. 6*a* ▸).

**Figure 7:**
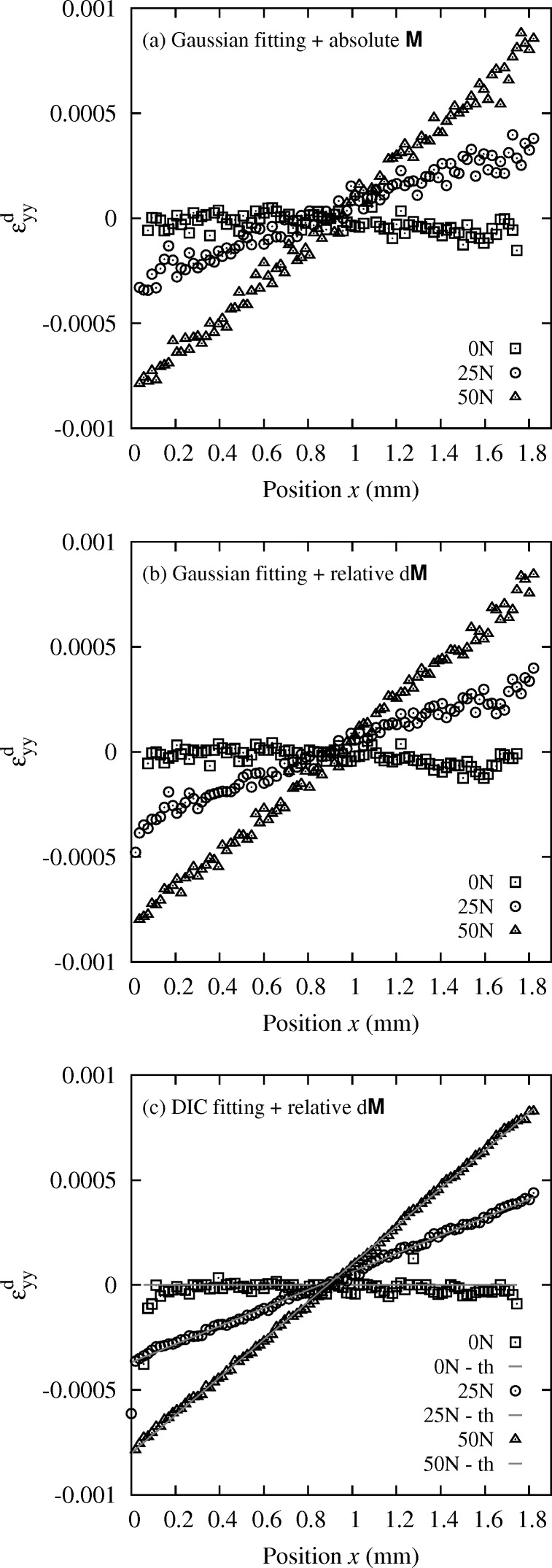
Profile of deviatoric elastic strain 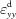 in the *in situ* bent Si crystal, along direction 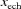, for three load levels: 0 N, 25 N, 50 N. (*a*) Absolute evaluation with the standard method described in §3.1. (*b*) Relative evaluation with the standard method. (*c*) Relative evaluation with the new Laue-DIC method. Comparison with the asymptotic beam theory donated by ‘th’.

Repeating this procedure for each 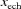, the distribution of deviatoric elastic strain can be plotted with a micrometric spatial resolution for the line scan along 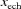 with a 20 µm step. Results are shown in Fig. 7(*c*) ▸. It can be observed that profiles of 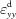 for an overall compression force on the bending setup of 25 N and 50 N are very close to linear, as expected from the asymptotic beam theory for four-point bending tests. The deviation of data from this linear trend is very small. Slightly larger data spread is observed for the 0 N profile; this point is discussed later.

For comparison, we report in Fig. 7(*a*) ▸ results obtained with the standard Laue method described in §3.1 for which the absolute Laue spots positions are determined by fitting, and compared with a calculated pattern of a known reference (Hofmann *et al.*, 2011 ▸; Hofmann, 2011 ▸). This was done with the *XMAS* software (Tamura *et al.*, 2002*c* ▸). The overall trend is still linear as expected, but data uncertainty is clearly larger than in Fig. 7(*c*) ▸ with the proposed Laue-DIC method. There are two main differences between the two methods. First, the standard method relies on a fitting of Laue spots by an analytical function; this introduces some errors in the peak position. Second, as seen above, the standard method measures 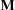 and not 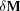, and 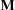 is highly sensitive to uncertainties on the geometry parameters. This method also requires knowing the stress-free parameters 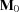 with high accuracy. Fig. 7(*b*) ▸ shows the 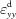 profiles obtained by minimizing equation (15) on the spot displacement field 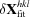 = 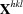 − 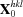, with 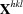 and 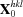 measured by Gaussian fitting of spot shape using the *XMAS* software (procedure denoted hereafter ‘relative method’). Whatever the uncertainty on 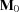, the comparison of Figs. 7(*c*) and 7(*b*) ▸ illustrates the gain brought by DIC in terms of accuracy on spot displacements, and consequently on strain components.

**Figure 8:**
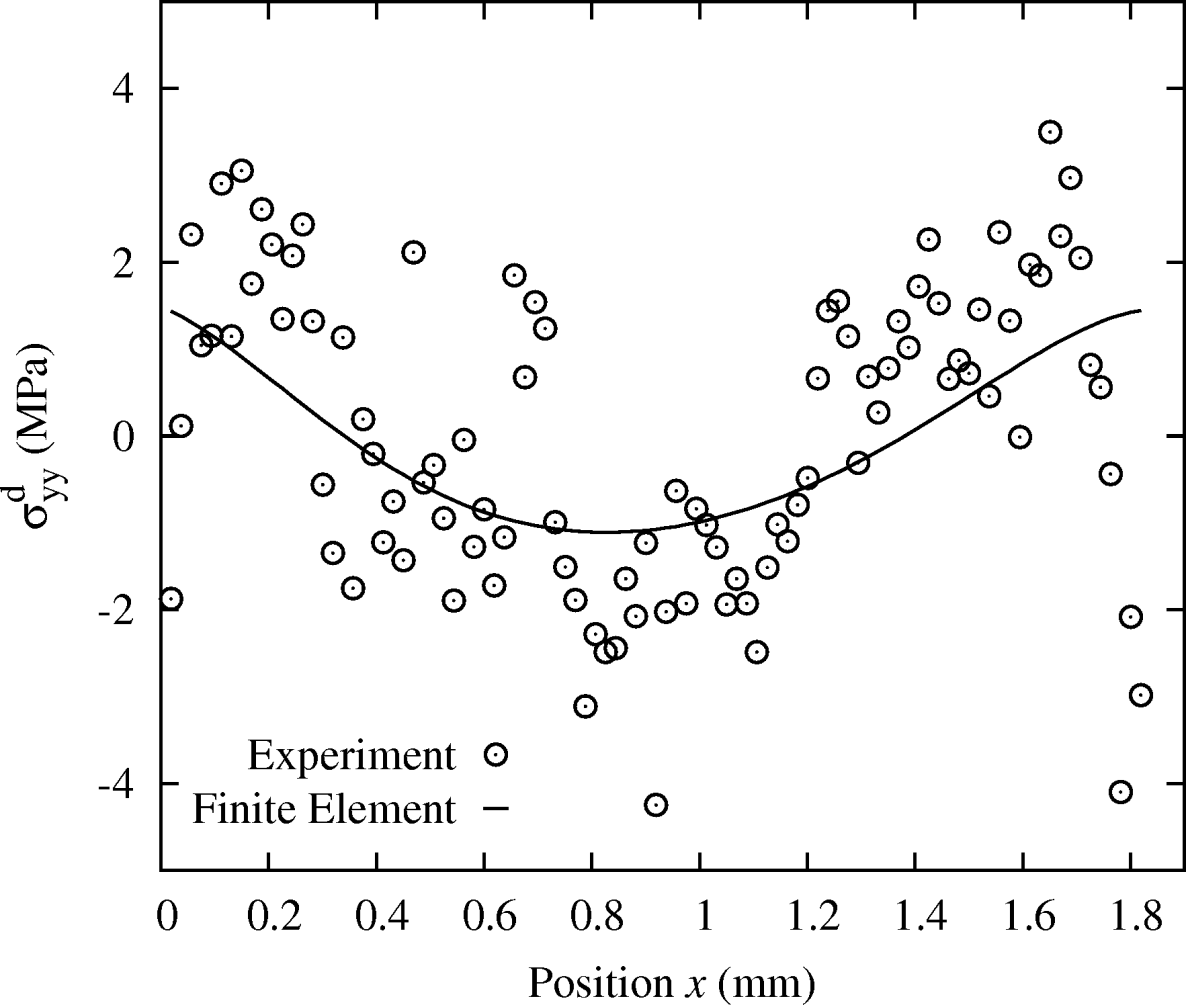
Evolution of 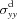 along 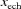 in which the linear trend of data, as observed in Fig. 10 ▸, has been substracted to highlight the slight nonlinearity. Experimental data are compared with FE results. Case 50 N.

In order to evaluate in a more quantitative way the errors associated with these procedures, a theoretical solution for the deformation of the specimen is required. When using the Laue-DIC method, the accuracy on the measured stress profiles starts to be sufficient to detect minor deviations with respect to the beam asymptotic theory. Such deviations are expected since the width and thickness of the sample are not negligible with respect to its length, and the crystal is elastically anisotropic. Fig. 8 ▸ shows the difference (black circles) between the 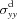 stress profile measured at 50 N and the linear 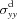 profile predicted by the beam asymptotic theory.

**Figure 9:**
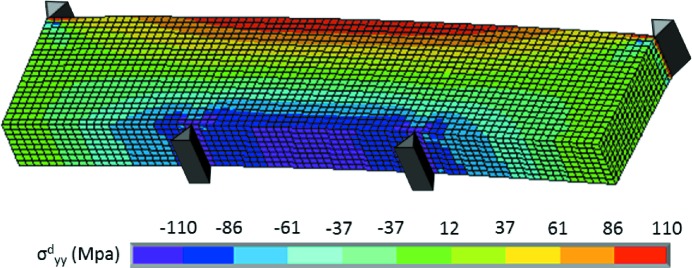
Distribution of 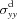 in the Si single-crystal deformed under four-point bending, obtained by FE modeling at 50 N. The anisotropic elastic constants of Si crystal and the actual crystal orientation have been used.

This deviation can be reproduced by a FE calculation that takes into account both the actual geometry and crystal orientation, and includes elastic anisotropy. The simulation was performed with the commercial code *ANSYS*. We used the anisotropic elastic constants for Si single-crystal (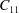 = 166.0 GPa, 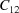 = 64.0 GPa and 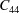 = 79.6 GPa using Voigt notation), and actual specimen and deformation rig geometries. As for the boundary conditions, nodal forces were applied on each of the four lines representing the loading pins, and one node of the structure was blocked in all directions to avoid rigid-body translations. Fig. 9 ▸ shows the distribution of 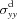 obtained with the FE model for a loading of 50 N.

**Figure 10:**
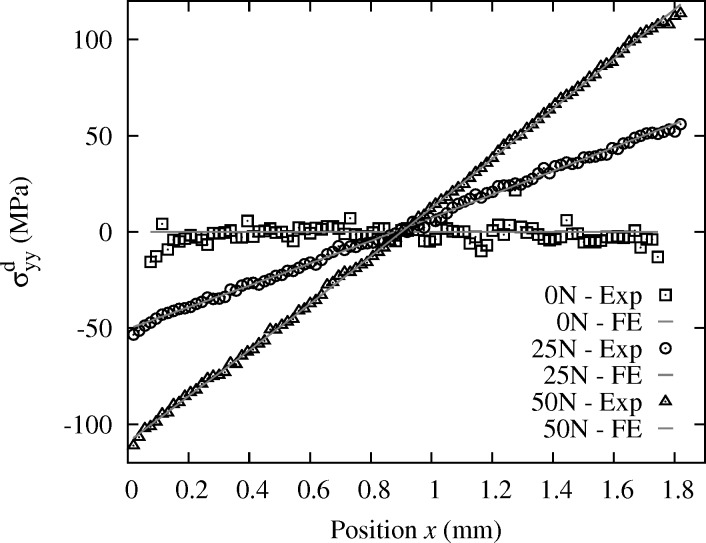
Longitudinal component 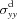 of the deviatoric stress obtained by Laue-DIC along the specimen thickness for the loadings 0 N, 25 N and 50 N. Corresponding values obtained by the FE model are also indicated.

In the case of a Si crystal, owing to the cubic symmetry of the crystal lattice, the components of the deviatoric stress tensor can be computed from the experimental deviatoric elastic strain, as explained in the supporting information. Experimental profiles of 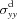 along axis 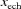 obtained by the new Laue-DIC method for the three loadings are plotted in Fig. 10 ▸. Corresponding data obtained by the FE model are also plotted. An excellent match between the experimental and numerical profiles is found. The general trend of those profiles is linear; however, a more careful look indicates a slightly non-linear evolution of 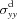 with 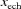. This feature becomes more clear after having subtracted the linear part of the 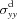 evolution from the experimental data and the FE simulation, as shown in Fig. 8 ▸ for the 50 N case. Again, very good agreement between experimental data and numerical results is obtained. We also verified that the fluctuations observed around the FE stress profile do not come from the coupling between normal and shear strain components. Indeed, this coupling is very weak: small coupling elastic constants (only 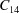, 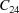, 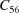 are non zero and less than 13.6 GPa) and small shear strain values. However, this latter could have more influence in terms of noise, in other orientations, because of larger uncertainties on shear strain (Poshadel *et al.*, 2012 ▸; Hofmann *et al.*, 2013 ▸).

A quantitative analysis of the accuracy of Laue-DIC results was performed by calculating the standard deviations of the discrepancy between experimental strain and stress profiles and results from the FE model; results are collected in Table 1 ▸. In addition, from the experimental deviatoric stress profile 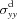, it is also possible to estimate the overall load applied to the specimen for the bending test, assuming that 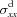 = 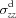 = 0 MPa along the 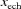 axis at 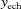 = 0 mm. Values of 24.3 N and 51.6 N were obtained (Table 1 ▸), *i.e.* a difference of only 2.8% and 3.2% with respect to the nominal loads (25 N and 50 N). This is consistent with the expected accuracy on the experimental value of the applied force, given the accuracy of the force sensor. Slightly worse results (Table 1 ▸) are obtained for 0 N than for 25 N and 50 N. A possible explanation could be that tiny specimen motions occur during the Laue scan at 0 N due to the difficulty in holding the small specimen under stress-free conditions.

The following conclusions can be thus drawn from the analysis of Table 1 ▸:

(i) Laue-DIC provides the best match to the FE reference solution, with a standard deviation two to four time smaller than the standard data treatment method (detailed in §3.1).

(ii) The main source of error of the standard method is the fitting of the Laue spot by an analytical function. Indeed, using the relative method as for Fig. 7(*b*) ▸ [column denoted (*b*) in Table 1 ▸] only slightly improves on the standard deviation as compared with the standard method. Comparison of the *residues* of the two relative evaluation methods confirms the fitting perfomance gained with DIC.

(iii) For the specimen investigated in this study, Laue spot shape does not deviate much from Gaussian (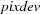 = 0.77 pixels while 0.2–0.3 pixels is obtained in the case of Ge), and even in this case Laue-DIC provides superior results. One can thus anticipate much greater improvements when the Laue spot shape is more complex, *e.g.* for specimens subjected to plastic deformation. DIC is also able to handle the change of shape of a spot between the two Laue patterns. Specific ‘connected’ shape functions will need to be built to take advantage of the usual similarity of shape of neighboring spots when spot shape is dominated by orientation gradients.

Future plans include checking the validity of these conclusions on other samples.

### 5.4. Minor components of the stress tensor: Laue-DIC case at 50 N   

**Figure 11:**
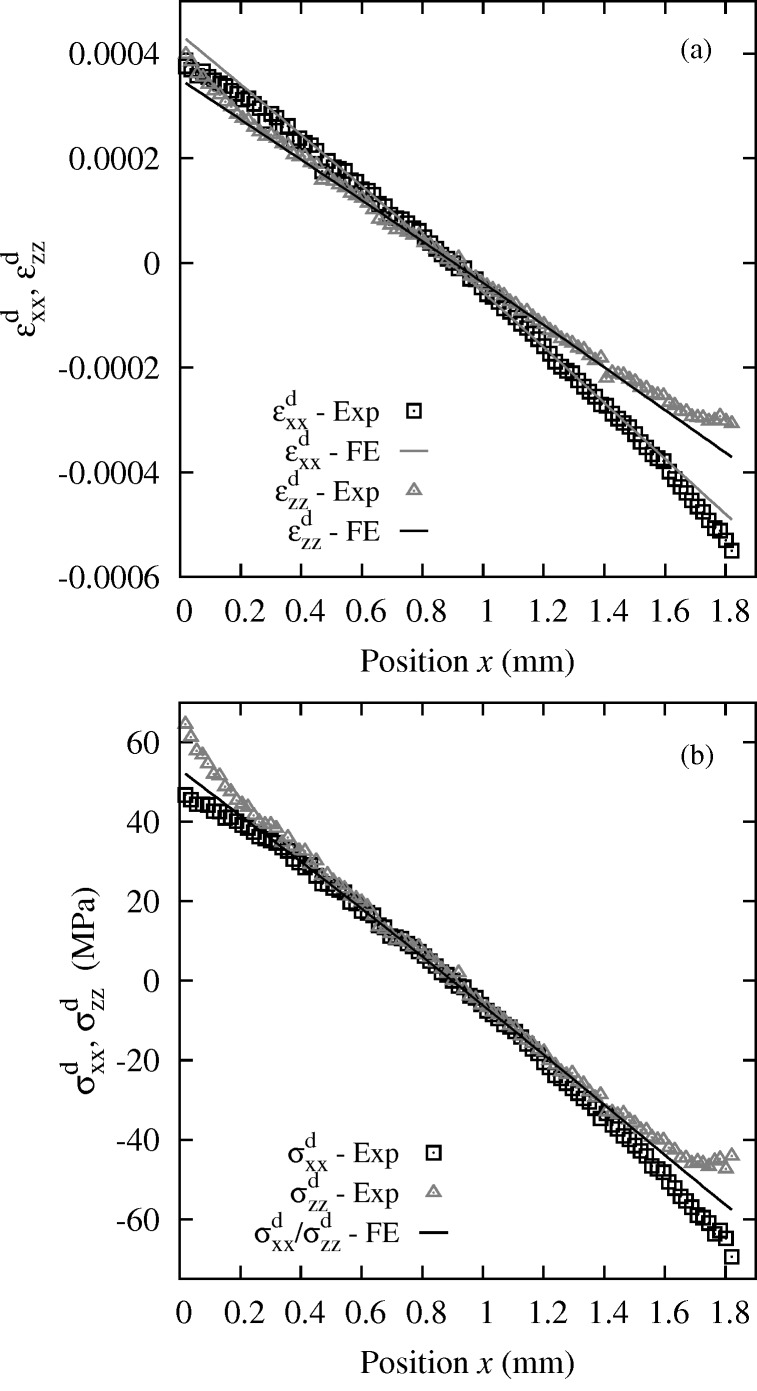
(*a*) Profile of the deviatoric strain components 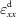 and 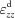 and (*b*) profile of the deviatoric stress components 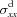 and 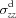, obtained by the Laue-DIC method on a Si single-crystal during bending at 50 N. In (*b*), the line referring to as ‘FE’ is the finite-element results for 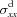 and 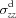.

Both methods provide all the components of the deviatoric stress tensor (either absolute or relative). Here, only the (more accurate) Laue-DIC analysis is shown. Evolution with 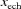 of the axial components 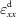 and 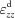 of the deviatoric strain and 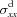 and 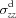 of the deviatoric stress are plotted in Fig. 11 ▸. The 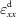 strain profile is not exactly linear, it is softly curved as predicted by the FE model. However, experimental curvature changes faster near the sample edges. In the same way, except edge effects, for 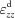, both experimental and numerical curves are almost linear and agree very well. The plots of the stress components are linear in the central part, *i.e.* next to the specimen neutral fiber, and deviate from linear at the edges near 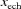 = 0 mm and 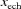 = 1.8 mm. However, in contrast to the 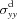 component, the FE calculation (solid line in Fig. 11 ▸) is unable to reproduce the experimental deviations. It can be checked that these effects are not compatible with free surface boundary conditions, which remains an issue of the present work. Such an effect is not fully understood at present and several possible reasons will be investigated in the future: (i) the test was not exactly a pure bending (*e.g.* due to some inaccuracy of the loading device); (ii) the anisotropy of the elastic properties together with the misalignment of the bending axis with the principal directions of the elastic properties generates a complex pattern of sample deformation; (iii) the sample surface was not really planar due to manufacturing and polishing, leading to artificial distortion of Laue patterns; (iv) an orientation error of the Si crystal or of the X-ray beam; (v) the free surface boundary conditions do not apply to Laue data due to the penetration of the X-ray beam; however, regarding this last hypothesis, FE stress profiles in depth along the X-ray beam were investigated and were found very similar to the FE stress profiles at the surface.

**Figure 12:**
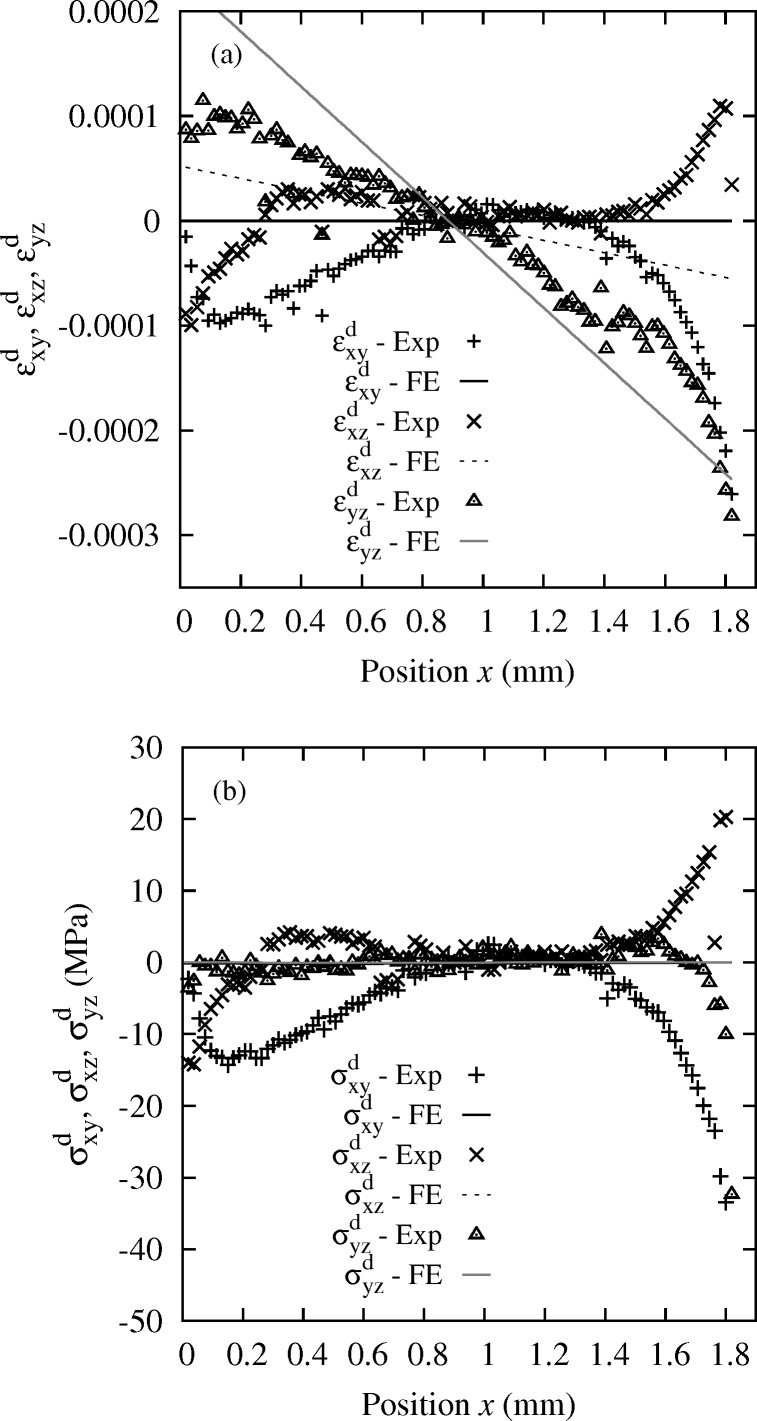
(*a*) Profile of the shear strain components 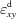, 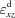 and 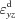 and (*b*) profile of the shear stress components 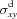, 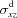 and 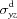 obtained by the Laue-DIC method for the Si single-crystal. Case 50 N.

Similarly, shear strain 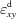, 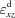, 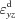 and shear stress components 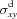, 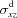, 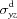 are plotted in Fig. 12 ▸. By comparison with the FE shear strain results, only for the 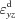 is the general tendency retrieved. For all shear components, large edge effects exist at the two ends. Shear stress components also become abnormally large at some 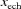 positions, which disagrees with the FE calculation that predicts ∼0 MPa for all three (lines in Fig. 12*b* ▸). Indeed, a pure bending test does not predict any shear stress and the free surface condition also imposes shear stresses with normal 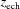 equal to 0 MPa. As for the normal components, this issue remains unexplained, and the possible reasons detailed just above could apply similarly here and need to be checked. However, it can be noted that mean values of the shear stress components along the 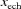 direction (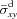 = −6.38 MPa, 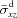 = 2.27 MPa, 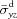 = −0.37 MPa) are rather close to those obtained by FE analysis. We have checked that the issues shown in Figs. 11 ▸ and 12 ▸ do not result from the Laue-DIC method; very similar features are also observed when applying the standard Laue method detailed in §3.1. In spite of the deviations at the edges, a significant portion of the stress profile between 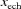 = 0.8 mm and 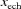 = 1.3 mm shows almost vanishing values for all components. Very similar features are observed when applying the standard method, except that the curves are more noisy. Furthermore, uncertainties on the ‘
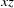
’ and ‘
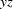
’ components, containing the out-of-plane direction 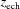, are systematically slightly larger than other components, as already seen and discussed by Poshadel *et al.* (2012 ▸), because of the limited reciprocal space coverage provided by the detector in our setup configuration.

### 5.5. Sensitivity to DIC parameters, and possible improvements   

Although we have shown above the superior results provided by the proposed Laue-DIC method and the excellent accuracy obtained for 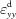, further improvements are still possible, as explained now. Finding the image transformation that provides the best correlation between the initial and deformed images can be largely influenced by the input parameters of the DIC algorithm.

We have thus investigated the sensitivity of DIC accuracy with respect to the size of the ZOI and the degree of the polynomial function used for the subpixel gray-level interpolation of the deformed image. Results are reported in Table 2 ▸. Globally, DIC accuracy decreases when increasing the ZOI size, and slightly better results are obtained when using interpolation functions of higher order. The effect of these two parameters is, however, relatively small; excellent results are already obtained with the fastest algorithm, *i.e.* 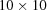 ZOI and bilinear interpolation. This corresponds to a ZOI size slightly smaller than the size of the largest spot; for example, the size of the square ZOI around the largest Laue spot in Fig. 1 ▸ is 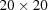
 pixels.

We have also tested an additional method in which the size and shape of the ZOI, considered rectangular, is fitted to the size of each individual spot, so that the ZOI contains the entire spot but as few background pixels as possible that do not contain any physical information on the diffraction process. It can be observed that such an optimized ZOI provides better results when bilinear and biquintic interpolations are used.

The goal of the DIC procedure is to find the image transformation 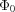 that one has to apply to the initial image to match as closely as possible the deformed image. In this work, the simplest image transformation was used, consisting of a sole translation. But more complex image transformation can be applied, such as translation and rotation or any higher-order transformation representing for example the spreading of a Laue spot with increasing strain. Such a more complex image transformation gave no improvement here, but could help in future work when dealing, for example, with plastic strain.

The number of spots taken into account when minimizing equation (15) significantly influences the determination of the transformation gradient 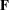. To investigate this effect, we have computed the stress standard deviation using from 34 to 75 spots. Spots were sorted by decreasing intensity, so that increasing the number of spots added only lower intensity spots. Results are reported in Table 3 ▸. It can be observed that increasing the number of spots leads to a smaller standard deviation, *i.e.* better results. In other words, although DIC applied to spots with low intensity often leads to a relatively large correlation coefficient (10), using such spots in the minimization procedure improves on the determination of the stress state.

**Figure 13:**
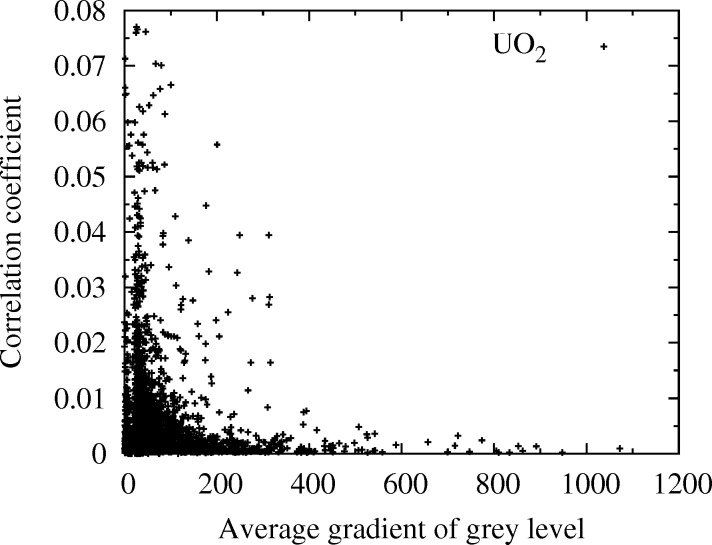
Correlation coefficient *C* *versus* the average gradient of the gray level in the ZOI of each spot. Specimen of UO_2_.

Finally, it is worth recalling that uncertainties on displacement measured by DIC decrease when increasing the mean gray-level contrast in the ZOI (see, for example, Roux & Hild, 2006 ▸). Hence, for a given shape function 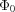, the higher the average gray-level gradient in the ZOI, the better the correlation coefficient *C*. This coefficient *C* indicates the degree of resemblance between a spot of the reference image and a spot in the deformed configuration. It is thus of interest to investigate the sensitivity of *C* with respect to the spot intensity. We have used for that a specimen of uranium oxide (UO_2_), comprising a very large number of spots. DIC was performed between two almost identical Laue patterns successively acquired under the same conditions at the same position on the specimen (and thus differing only from the image noise), and the average gray-level gradient in the ZOI of each spot of the reference image has been computed by a finite differences method (the average gray-level gradient increases with the spot intensity). Results are shown in Fig. 13 ▸. It is found that a small correlation coefficient is obtained systematically for spots with large gray-level gradients (*i.e.* the intense spots). Spots exhibiting a small gradient (*i.e.* smaller intensity) generally give rise to larger correlation coefficient, *i.e.* a less accurate measurement of displacement by DIC. Thus, a future possible improvement of the Laue-DIC procedure could be to favor intense spots in the minimization of equation (15), *e.g.* by allocating them a larger weight than low-intensity spots.

## 6. Conclusion   

In this work, we have proposed a new Laue-DIC method based on the coupling between white-beam Laue microdiffraction and DIC techniques. The method is suitable for determining the Laue spot displacement field between two different deformation/orientation states and for deducing the associated increment of local strain and hence stress, with micrometer spatial resolution. The procedure can be decomposed into four steps:

(i) First, a Laue pattern is indexed from the known crystal structure, for example using the *LaueTools* software. The crystal orientation in the initial configuration can be estimated.

(ii) Next, DIC technique is used to determine the spot motion field on the detector between the initial and the deformed configurations.

(iii) Finally, a cost function minimization method is used to evaluate the mechanical transformation between the two configurations.

(iv) When possible (see the supporting information), the deviatoric stress can be computed from the measured deviatoric elastic strain. Indeed, as shown in this note, the local constitutive relation 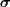 = 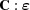 can be transformed into 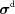 = 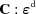 only when one deals with material exhibiting local isotropic elasticity 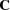 or for crystals with a cubic crystal lattice. In all other cases, one cannot evaluate the stress tensor (or its deviatoric part) if only the deviatoric elastic strain is known.

An important part of this paper was dedicated to the evaluation of the accuracy of the Laue-DIC procedure. A specimen exhibiting a simple and known microstructure (Si single-crystal) was deformed *in situ* in a controlled way, so that the measured strain distribution in the specimen could be compared with the reference distribution computed by FE. This approach allowed us to conclude that local normal stress along the specimen length direction under pure bending is estimated with high accuracy: the standard deviation of the error on 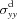, compared with a FE model, is found to be ∼1 MPa in the considered Si single-crystal deformed under four-point bending. Larger differences have, however, been obtained on other components of the deviatoric stress tensor, even though a comparable accuracy with 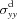 is reached far from the edges. These differences are not believed to be due to the Laue-DIC method itself but rather to experimental difficulties as explained above. Newer data are now needed to investigate this feature in more detail.

Thanks to the sub-micrometer size of the X-ray beam, stress field heterogeneities can be detected with a micrometric resolution. The method can thus be applied for measuring the stress field in deformed polycrystals with a spatial resolution smaller than the grain size. We have also provided a few possible directions for further improvement of the new Laue-DIC method, *e.g.* by weighting Laue spots according to their intensity gradient during the identification step.

## Supplementary Material

Relation between deviatoric stress and deviatoric strain. DOI: 10.1107/S1600577515005780/co5059sup1.pdf

## Figures and Tables

**Figure 14:**
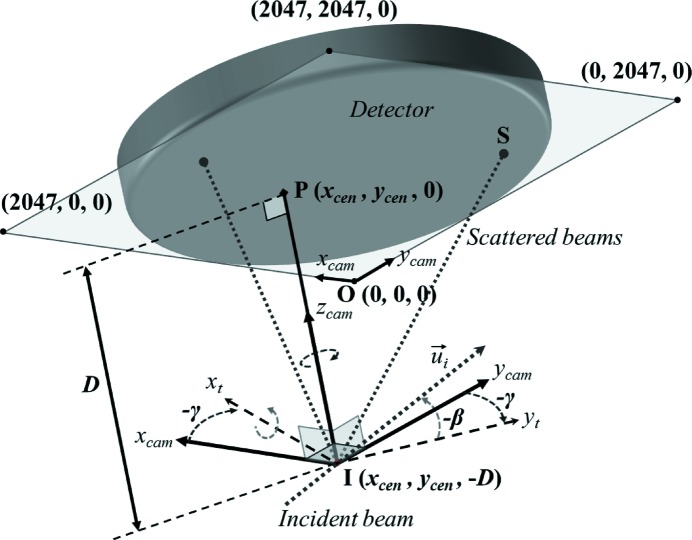
Scheme of the setup: orientation and position of the X-ray incoming beam 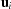 in the detector and camera reference frames, definition of the five geometrical calibration parameters. Point P is the normal projection on the detector screen of the impact point I of X-rays on the specimen surface.

**Table 1:** Standard deviations (SD) on experimental strain and stress evolutions with respect to the FE results, for loadings of 0 N, 25 N and 50 N, obtained for different treatment methods of Laue images: (*a*) standard − absolute, (*b*) standard − relative, (*c*) Laue-DIC. The computed applied load that can be estimated from the measured profile of deviatoric stress is also indicated. Units are µm m^−1^, MPa and N. The second part of the table gives the residue of equation (15), and *pixdev*, both in pixel units (*pixdev* is the average deviation between the measured Laue spot positions and the theoretical ones)

	(*a*)	(*b*)	(*c*)	(*c*)	
Experimental load	SD( 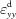 )	SD( 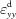 )	SD( 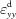 )	SD( 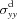 )	Computed load
0 N	50.4	44.9	27.5	3.65	–
25 N	41.0	35.9	10.2	1.51	24.3
50 N	41.9	34.8	9.6	1.38	51.6
					
*pixdev*	0.77	0.77	–	–	
Residue	–	0.22	0.05	0.05	

**Table 2:** Stress standard deviation SD(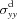) obtained for various degrees of the gray-level interpolation function and sizes of the ZOI (expressed in pixels). Column ‘Opt.’ reports results for a rectangular ZOI whose size is optimized to match the spot spread

	10 × 10	20 × 20	30 × 30	40 × 40	50 × 50	Opt.
Bilinear	1.43	1.65	1.49	1.46	1.55	1.38
Bicubic	12.69	13.19	13.66	14.41	17.21	14.61
Spline bicubic	12.77	13.26	13.84	14.12	17.37	14.79
Biquintic	1.31	1.31	1.35	1.38	2.50	1.30

**Table 3:** Effect of the number of spots taken for minimizing (15) on the stress standard deviation SD(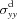). Results obtained for a ZOI size adapted to match the Laue spot size, and for different gray-level interpolation functions

	34	43	66	71	75
Bilinear	1.75	1.63	1.40	1.43	1.38
Bicubic	18.36	15.73	16.02	14.71	14.61
Spline bicubic	18.26	15.70	16.16	14.89	14.79
Biquintic	1.58	1.51	1.40	1.36	1.30

## Appendix A Geometrical setup

The reference frame,   used in this work is attached to the detector, as the detector remains fixed during the experiment (Fig. 14 ▸). The origin *O* of this reference frame is taken at an edge of the detector surface, and vector  is perpendicular to the detector screen, pointing from the sample to the detector. At BM32, the incident X-ray beam (unit vector ) lies almost parallel to the detector surface, and therefore we take  close to . Two small angles β and γ are needed to express  in the detector frame,

The position of the diffracting volume (point I in Fig. 14 ▸) is given by its three coordinates ( = −*D*), where  and  are usually expressed in pixel units and  = −*D* in millimeters. We are therefore left with five calibration parameters (β, γ, ,  and *D*). Note that this geometrical description of the setup is only approximative, since the real diffracting volume has an extended shape along the incident beam direction (due to absorption effects), the length of which may vary from spot to spot as the penetration of the beam depends on the X-ray energy (which differs from spot to spot). Any spot position on the detector screen is expressed by  and  in (decimal) pixel units, in the range [0, 2047] (the third coordinate of any point lying on the detector surface is 0 in the detector frame). Note also that a sample frame  =  is defined on Fig. 5(*a*) ▸, as the detector frame translated by −*D* along  and rotated by 40° around .

## Appendix B Calibration function

We provide in this Appendix details about function *f* appearing in equation (4), that provides the two coordinates of the Laue spot on the detector screen for a given strained orientation matrix  and a diffracting plane with Miller indices . The function *f* depends on all five calibration parameters defined in Appendix *A* [App appa], namely , , *D*, β and γ. Note that  may potentially vary with *hkl*, *i.e.* from spot to spot, and from one sample point to another (in case, for example, of imperfect sample alignement, sample roughness, inhomogeneous crystal quality, inhomogeneous sample absorption), and between the sample of interest and the Ge reference sample. For convenience, the detector frame defined in Appendix *A* [App appa] will be chosen as the reference frame.

First, let ,  and  denote the three reciprocal lattice vectors

with *V* the volume of the crystal lattice given by the absolute value of the determinant of , and × the cross product. The unit vector  normal to the  diffracting plane (*i.e.* a vector parallel to the diffraction vector) reads

and the corresponding unit vector  parallel to the diffracted beam is given by

with  the unit vector parallel to the incoming beam. Denoting *S* the impact point of the diffracted beam on the detector surface (*i.e.* *S* provides the Laue spot position), vector  is given by

Function *f* expresses the  and  coordinates of point *S* in the detector reference frame,

with  and  the first two coordinates of vector  in the same frame.
