# Supplementary material for: Laue-DIC: a new method for improved stress field measurements at the micrometer scale
Source: J Synchrotron Radiat. 2015 May 9;22(Pt 4):980–94. doi: 10.1107/S1600577515005780 (PMC4787029; doi:10.1107/S1600577515005780)
Supplement: Supplementary file 1 [file s-22-00980-sup1.pdf]

JOURNAL OF
SYNCHROTRON
RADIATION

Volume 22 (2015)

Supporting information for article:

Laue-DIC: a new method for improved stress field measurements at the micrometer scale

J. Petit, O. Castelnau, M. Bornert, F. G. Zhang, F. Hofmann, A. M. Korsunsky, D. Faurie, C. Le Bourlot, J. S. Micha, O. Robach and O. Ulrich

Laue-DIC: a new method for improved stress field measurements at the micron scale

J. Petit,[a]* O. Castelnau,[b] M. Bornert,[c] F.G. Zhang,[b] F. Hofmann,[d] A.M. Korsunsky,[d] D. Faurie,[e] C. Le Bourlot,[f] J.S. Micha,[g,h,i] O. Robach[g,i,j] and O. Ulrich[g,i,j]

[a]LEME, Université Paris Ouest, 50 rue de Sèvres, F-92410 Ville d'Avray, France, [b]PIMM, CNRS, Arts & Métiers ParisTech, 151 Bd de l'hopital, F-75013 Paris, France, [c]Laboratoire NAVIER, Université Paris-Est, École des ponts ParisTech, F-77455 Marne-la-Vallée, France, [d]Department of Engineering Science, University of Oxford, Parks Road, Oxford, OX1 3PJ, U.K., [e]LSPM, CNRS, Université Paris 13, 93430 Villetaneuse, France, [f]INSA-Lyon, MATEIS CNRS UMR5510, F-69621 Villeurbanne, France, [g]Univ. Grenoble Alpes, INAC-SPrAM, F-38000 Grenoble, France, [h]CNRS, SPrAM, F-38000 Grenoble, France, [i]CRG-IF BM32 at ESRF, 71, Avenue des Martyrs, F-38000 Grenoble, France, and [j]CEA, INAC-SP2M, F-38000 Grenoble, France.

E-mail: johannpetit@u-paris10.fr

Supplementary material :

Relation between deviatoric stress and deviatoric strain

When using white beam Laue microdiffraction, only the *deviatoric* part of the strain tensor can be retrieved from Laue patterns, since the volume of the crystal lattice is not measured. In this appendix, we show that this limitation prevents measurement of the deviatoric stress tensor when dealing with poorly symmetric crystal lattices. Only for cubic crystals, or for materials for which the elastic stiffness tensor **C** is isotropic, can the deviatoric stress be estimated.

Let σ and ε be the stress and elastic strain tensors. The *deviatoric* stress and strain, $\sigma^{\mathbf{d}}$ = dev(σ) and $\varepsilon^{\mathbf{d}}$ = dev(ε) respectively, read

$$\sigma^{\mathbf{d}} = \mathbf{K} : \boldsymbol{\sigma}\,, \quad \varepsilon^{\mathbf{d}} = \mathbf{K} : \boldsymbol{\varepsilon} \tag{1}$$

or, using the Einstein convention for repeated indexes, $\sigma^d_{ij} = K_{ijkl}\sigma_{kl}$ and $\varepsilon^d_{ij} = K_{ijkl}\varepsilon_{kl}$. Here, $\mathbf{K}$ is the deviatoric projector, *i.e.* a fourth-order tensor that extracts the deviatoric part of any symmetrical second order tensor; it reads

$$\mathbf{K} = \mathbf{I} - \mathbf{J}\,, \quad I_{ijkl} = \frac{1}{2}(\delta_{ik}\delta_{jl} + \delta_{il}\delta_{jk})\,, \quad J_{ijkl} = \frac{1}{3}\delta_{ij}\delta_{kl}\,, \tag{2}$$

with δ_{ij} the Kronecker delta ($\delta_{ij} = 1$ if $i = j$, $\delta_{ij} = 0$ if $i \neq j$). The elastic constitutive relation reads

$$\sigma = \mathbf{C} : \varepsilon \tag{3}$$

with $\mathbf{C}$ the elastic stiffness tensor. When multiplied by tensor $\mathbf{K}$, one obtains

$$\sigma^d = \mathbf{K} : \boldsymbol{\sigma} = \mathbf{K} : \mathbf{C} : \boldsymbol{\varepsilon}\,, \quad \text{or} \quad \sigma^d_{ij} = K_{ijkl}C_{klmn}\varepsilon_{mn} \tag{4}$$

showing that the deviatoric stress σ^d can be derived from the deviatoric elastic strain ε^d only if

$$\mathbf{K} : \mathbf{C} = \mathbf{C} : \mathbf{K}\,. \tag{5}$$

Only under such a condition can equation (4) be writen

$$\sigma^d = \mathbf{C} : \varepsilon^d\,. \tag{6}$$

Note that both $\mathbf{K}$ and $\mathbf{C}$ are symmetrical tensors, but this does not imply that the product $\mathbf{K} : \mathbf{C}$ is symmetric as well. We detail below the consequences of condition (5) for different symmetries of the elastic stiffness, generally associated with the symmetry of the crystal lattice.

1. In case of *elastic isotropy*, the elastic compliance reads

$$\mathbf{C} = 3k\mathbf{J} + 2\mu\mathbf{K} \tag{7}$$

   with k and μ the compressibility and shear modulus respectively. Since $\mathbf{J} : \mathbf{K} = \mathbf{K} : \mathbf{J} = \mathbf{0}$ and $\mathbf{K} : \mathbf{K} = \mathbf{K}$, condition (5) is fulfilled and equation (6) applies.

2. For *cubic symmetry*, it is convenient to introduce tensors $\mathbf{\Lambda} = \mathbf{e}_1 \otimes \mathbf{e}_1 \otimes \mathbf{e}_1 \otimes \mathbf{e}_1 + \mathbf{e}_2 \otimes \mathbf{e}_2 \otimes \mathbf{e}_2 \otimes \mathbf{e}_2 + \mathbf{e}_3 \otimes \mathbf{e}_3 \otimes \mathbf{e}_3 \otimes \mathbf{e}_3$ with $\mathbf{e}_1$, $\mathbf{e}_2$, and $\mathbf{e}_3$ three unit vectors aligned with the directions of the cubic symmetry ($\otimes$ is the dyadic product), $\mathbf{K}^a = \mathbf{\Lambda} - \mathbf{J}$,

and $\mathbf{K}^b = \mathbf{K} - \mathbf{K}^a = \mathbf{I} - \mathbf{\Lambda}$. The following general expression holds for the stiffness tensor $\mathbf{C}$

$$\mathbf{C} = 3k\mathbf{J} + 2\mu^a\mathbf{K}^a + 2\mu^b\mathbf{K}^b\,. \tag{8}$$

Since $\mathbf{K}^a : \mathbf{K} = \mathbf{K} : \mathbf{K}^a = \mathbf{K}^a$ and $\mathbf{K}^b : \mathbf{K} = \mathbf{K} : \mathbf{K}^b = \mathbf{K}^b$, condition (5) is fulfilled and equation (6) applies.

3. For *general anisotropy*,

$$\mathbf{K} : \mathbf{C} = (\mathbf{I} - \mathbf{J}) : \mathbf{C} = \mathbf{C} - \mathbf{J} : \mathbf{C} \tag{9}$$

and therefore one only has to evaluate the symmetry of $\mathbf{J} : \mathbf{C}$. Noting that

$$J_{ijmn}C_{mnkl} = \frac{1}{3}\delta_{ij}C_{mmkl} \quad \text{and} \quad C_{ijmn}J_{mnkl} = \frac{1}{3}\delta_{kl}C_{ijmm} \tag{10}$$

and with the major symmetry of $\mathbf{C}$ (*i.e.* $C_{ijkl} = C_{klij}$), one has thus to check under which conditions

$$\delta_{ij}C_{mmkl} = \delta_{kl}C_{mmij} \quad \forall i, j, k, l \tag{11}$$

(with implicit summation on m).

(a) If $i \neq j$ and $k \neq l$, condition (11) is trivially fulfilled.

(b) If $i = j$ and $k \neq l$, or $i \neq j$ and $k = l$, condition (11) leads to $C_{11kl} + C_{22kl} + C_{33kl} = 0\ \forall k \neq l$. This is realized for full isotropy, transverse isotropy, and for cubic, orthotropic, quadratic, and rhomboedric symmetries, not for monoclinic and triclinic materials.

(c) If $i = j$ and $k = l$, condition (11) reads $C_{11ii} + C_{22ii} + C_{33ii} = C_{11kk} + C_{22kk} + C_{33kk}\ \forall i, k$ (with no summation on i and k). This is realized only in case of complete isotropy and cubic symmetry.

In summary, the local constitutive relation $\sigma = \mathbf{C} : \varepsilon$ can be transformed into $\sigma^d = \mathbf{C} : \varepsilon^d$ only when one deals with material exhibiting local isotropic elasticity $\mathbf{C}$ or for crystals with a cubic crystal lattice. In all other cases, one cannot evaluate the stress tensor (nor its deviatoric part) from the only knowledge of the deviatoric elastic strain. As an illustrative example, one can consider the case of a triclinic lattice, for which the stiffness tensor $\mathbf{C}$

has 18 independent coefficients; it is easy to verify that all shear stress components depend on the trace of the elastic strain, which is unknown.
